# Scalable Design of Paired CRISPR Guide RNAs for Genomic Deletion

**DOI:** 10.1371/journal.pcbi.1005341

**Published:** 2017-03-02

**Authors:** Carlos Pulido-Quetglas, Estel Aparicio-Prat, Carme Arnan, Taisia Polidori, Toni Hermoso, Emilio Palumbo, Julia Ponomarenko, Roderic Guigo, Rory Johnson

**Affiliations:** 1 Centre for Genomic Regulation (CRG), The Barcelona Institute for Science and Technology, Dr. Aiguader 88, Barcelona, Spain; 2 Universitat Pompeu Fabra (UPF), Barcelona, Spain; 3 Institut Hospital del Mar d’Investigacions Mediques (IMIM), Barcelona, Spain; 4 Department of Clinical Research, University of Bern, Bern, Switzerland; 5 Department of Medical Oncology, Inselspital, University Hospital and University of Bern, Bern, Switzerland; Ottawa University, CANADA

## Abstract

CRISPR-Cas9 technology can be used to engineer precise genomic deletions with pairs of single guide RNAs (sgRNAs). This approach has been widely adopted for diverse applications, from disease modelling of individual loci, to parallelized loss-of-function screens of thousands of regulatory elements. However, no solution has been presented for the unique bioinformatic design requirements of CRISPR deletion. We here present CRISPETa, a pipeline for flexible and scalable paired sgRNA design based on an empirical scoring model. Multiple sgRNA pairs are returned for each target, and any number of targets can be analyzed in parallel, making CRISPETa equally useful for focussed or high-throughput studies. Fast run-times are achieved using a pre-computed off-target database. sgRNA pair designs are output in a convenient format for visualisation and oligonucleotide ordering. We present pre-designed, high-coverage library designs for entire classes of protein-coding and non-coding elements in human, mouse, zebrafish, *Drosophila melanogaster* and *Caenorhabditis elegans*. In human cells, we reproducibly observe deletion efficiencies of ≥50% for CRISPETa designs targeting an enhancer and exonic fragment of the *MALAT1* oncogene. In the latter case, deletion results in production of desired, truncated RNA. CRISPETa will be useful for researchers seeking to harness CRISPR for targeted genomic deletion, in a variety of model organisms, from single-target to high-throughput scales.

## Author Summary

CRISPR-Cas9 is a revolutionary biological technique for precisely editing cells’ genomes. Amongst its many capabilities is the deletion of defined regions of DNA, creating a wide range of applications from modelling rare human diseases, to performing very large knock-out screens of candidate regulatory DNA. CRISPR-Cas9 requires researchers to design small RNA molecules called sgRNAs to target their region of interest. A large number of bioinformatic tools exist for this task. However, CRISPR deletion requires the design of optimised *pairs* of such RNA molecules. This manuscript describes the first pipeline designed to accomplish this, called CRISPETa, with a range of useful features. We use CRISPETa to design comprehensive libraries of paired sgRNA for many thousands of target regions that may be used by the scientific community. Using CRISPETa designs in human cells, we show that predicted pairs of sgRNAs produce the expected deletions at high efficiency. Finally, we show that these deletions of genomic DNA give rise to correspondingly truncated RNA molecules, supporting the power of this technology to create cells with precisely deleted DNA.

## Introduction

CRISPR/Cas9 is a simple and versatile method for genome editing that can be applied to deleting virtually any genomic region for loss-of-function studies. Deletion requires the design of optimal pairs of single guide RNA (sgRNA) molecules that hybridise to sequences flanking the target region. While this approach is being employed for diverse applications, from single target studies [1–3] to high parallelized screening studies [4,5], there presently exists no bioinformatic solution for selection of optimal pairs of sgRNAs. We present here a highly customisable design pipeline to address the needs of all such deletion projects, regardless of scale.

CRISPR/Cas9 makes it possible to investigate the function of genomic elements in their endogenous genetic context. The Cas9 nuclease is recruited to desired genomic sites through its binding to an engineered, single guide RNA (sgRNA) [6]. Early studies focussed on protein coding genes, utilizing individual sgRNAs to induce small indel mutations in genomic regions encoding target proteins’ open reading frame (ORFs). Such mutations frequently give rise to inactivating frameshift mutations, resulting in complete loss of function [7,8]. The delivery of a single sgRNA in such experiments is technically straightforward, and can be scaled to genome-wide, virally-delivered screens.

CRISPR has also been brought to bear on non-coding genomic elements, including regulatory regions and non-coding RNAs, which have traditionally resisted standard RNA interference (RNAi) [2,9]. With some exceptions (for example [10]), functional knockout of non-coding elements with a single sgRNA is not practical, because small indel mutations caused by single sgRNAs are less likely to ablate function to the same extent as in a protein-coding sequence. Instead, a deletion strategy has been pursued: a pair of sgRNAs are used to recruit Cas9 to sites flanking the target region [2,4]. Simultaneous strand breaks are induced, and non-homologous end joining (NHEJ) activity repairs the lesion. In a certain fraction of cases, this results in a genomic deletion with a well-defined junction [4].

Cas9 targeting is achieved by engineering the 5’ variable region of the sgRNA. This hybridises to a complementary “protospacer” region in DNA, immediately upstream of the “protospacer adjacent motif” (PAM) [11]. For the most commonly-used *S*. *pyogenes* Cas9 variant, the PAM sequence consists of “NGG”. A growing number of software tools are available for the selection of optimal individual protospacer targeting sequences [12–18]. The key selection criteria are (1) the efficiency of a given sequence at generating mutations, and (2) “off-targeting”, or the propensity for recognising similar, yet undesired, sites in the genome. Based on experimental data, scoring models for on-target efficiency have been developed, for example that presented by Doench et al [16]. At the same time, tools have become available for identifying unique sgRNA sites genome-wide, mitigating to some extent the problem of off-targeting [19]. However, few tools presented so far are designed for large-scale designs, and to the best of our knowledge, none was created to identify optimal sgRNA *pairs* required for deletion studies.

To address this need, we here present a new software pipeline called CRISPETa (CRISPR Paired Excision Tool) that selects optimal sgRNAs for deletion of user-defined target sites. The pipeline has several useful features: first, it can be used for any number of targets in a single, rapid analysis; second, it returns multiple, optimal pairs of sgRNAs, with maximal predicted efficiency and minimal off-target activity; third, the user has control over the full range of design parameters. The pipeline is available as both standalone software and as a user-friendly webserver. In addition, we make available a number of pre-designed deletion libraries for various classes of non-coding genomic elements in a variety of species. Finally, using a quantitative deletion assay, we find that CRISPETa predictions are highly efficient in deleting fragments of a human gene locus, resulting in detectable changes to the cellular transcriptome. CRISPETa is available at http://crispeta.crg.eu.

## Results

### The CRISPETa pipeline for paired sgRNA design

**Fig 1:**
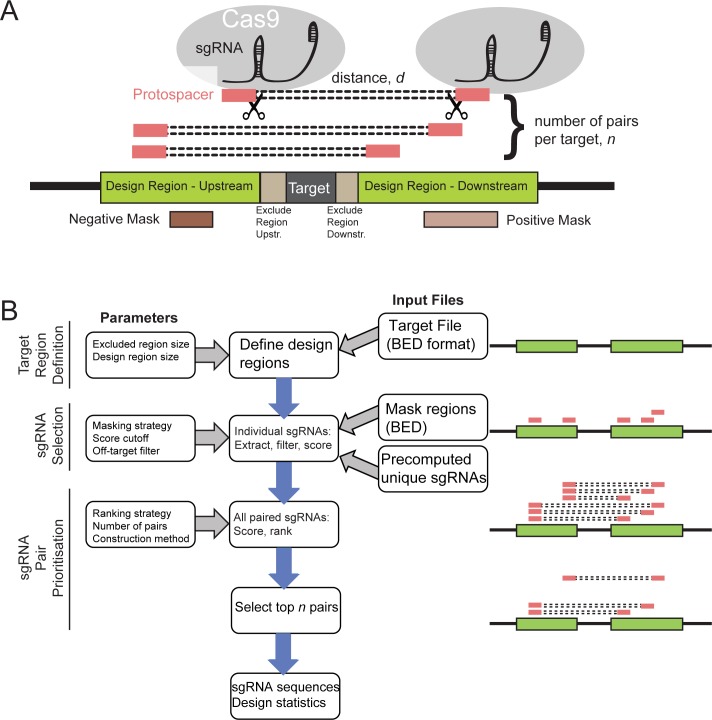
Overview of CRISPETa pipeline. (A) Schematic of CRISPR-mediated genomic deletion. The aim is elimination of the Target region through recruitment of a pair of Cas9 proteins. Red boxes represent protospacers, the 20 bp upstream of a PAM and recognised by the sgRNA. (B) The CRISPETa workflow.

To address the need for bioinformatic design of paired sgRNAs for genomic deletion, we created the CRISPETa pipeline (Fig 1A and 1B). The guiding principles of CRISPETa are flexibility and scalability: the user has control over all aspects of the design process if desired (otherwise reasonable defaults are provided), and the design may be carried out on individual targets, or target libraries of essentially unlimited size. The full set of user-defined variables, and their default values, are shown in Table 1.

**Table 1:** User-defined parameters.

Parameter	Symbol	Default	Comments
Input file	*i*	Mandatory	Path to input BED file.
Genome	*g*	Mandatory	Path to genome in FASTA format.
Off Targets	*t*	1,0,0,x,x	String with maximum number of off-targets allowed with 0,1,2,3 and 4 mismatches (x: no limit).
Output prefix	*o*	“sgRNA_pairs”	Path/prefix of output files.
Number of sgRNA pairs per target	*n*	10	Maximum number of pairs to be returned.
Upstream design region (bp)	*du*	500 bp	Length of upstream region for protospacer search.
Downstream design region (bp)	*dd*	500 bp	Length of downstream region for protospacer search.
Upstream exclude region (bp)	*eu*	100 bp	Length of upstream region adjacent to target excluded from protospacer search.
Downstream exclude region (bp)	*ed*	100 bp	Length of downstream region adjacent to target excluded from protospacer search.
Diversity	*v*	0.5	The maximum fraction of returned pairs that contain the same protospacer.
Individual score	*si*	0.2	The minimum score individual protospacers must have to be considered.
Paired score	*sp*	0.4	The minimum combined score that a protospacer pair must have to be considered.
Score combination	*sc*	sum	Method by which individual scores are combined to yield pair score: addition (“sum”) or multiplied (“product”).
Ranking method	*r*	score	Criteria for ranking protospacer pairs (“score” or “distance”).
Construct method	*c*	none	Method applied when making protospacer pairs and oligo construction: “none” or “DECKO” (first protospacer starts with G)
Positive mask	*mp*	-	Favoured regions from genome, in BED format.
Negative mask	*mn*	-	Disfavoured regions from Genome, in BED format.

The core objective of sgRNA design is the selection of optimal “protospacers”, defined as the 20 bp of genomic DNA sequence preceding the PAM sequence [11]. This is distinct from the sgRNA sequence itself, composed of the protospacer sequence and the constant, scaffold region (Fig 1A).

The CRISPETa workflow is divided into three main steps: target region definition, protospacer selection, and sgRNA pair prioritisation (Fig 1B). Given a genomic target region or regions in BED format, CRISPETa first establishes pairs of “design regions” of defined length in which to search. Design regions may be separated from the target itself by “exclude regions” of defined length. The user may also specify “mask regions”: sgRNAs falling within the positive mask are prioritised, whereas those within the negative mask will be de-prioritized (although not removed altogether). Positive masks might include regions of DNaseI-accessible chromatin, while negative masks may be composed of, for example, repetitive regions or compact chromatin.

**Fig 2:**
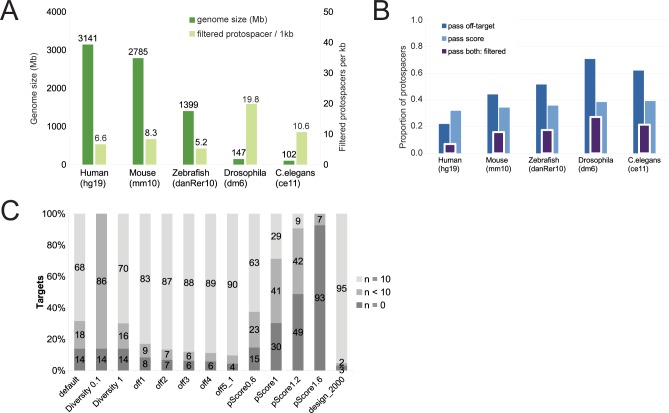
Benchmarking and performance. (A) Genome size and filtered protospacer density for the five species tested. (B) The fraction of protospacers passing filters of off-targeting, efficiency score, and both. The latter are defined as “filtered protospacers”, whose density is shown in (A). Data are displayed as a fraction of the total number of canonical PAM sequences in each genome. (C) The effect on library quality of modifying design variables. Y-axis denotes the percent of target regions, divided by: “successful”, where n = 10 distinct sgRNA pair designs are returned per target; “intermediate” designs, where 0<n<10 pairs are returned; “failed” designs, where n = 0 pairs are returned. CRISPETa was run on a test set of 7000 targets (see Materials and Methods for details). The first column represents the run performed with default settings, and in each subsequent column one variable is modified (see Table 3 for details).

Using this information, the entire set of potential protospacers is defined. First, the design region sequence is extracted and searched for all possible 20mer sites followed by canonical *S*. *pyogenes* “NGG” PAM sites–candidate protospacers. These are considered with respect to two core metrics: their potential for off-target binding, and their predicted efficiency. Off-targeting, or the number of identical or similar sites with a given number of mismatches, is estimated using precomputed data for each genome. This strategy increases the speed of CRISPETa dramatically. We created off-target databases for five commonly-studied species, human, mouse, zebrafish, *Drosophila melanogaster*, *Caenorhabditis elegans* (Table 2), varying widely in genome size (Fig 2A). The default off-targeting cutoff is set at (0:1, 1:0, 2:0, 3:x, 4:x), that is, sequences having no other genomic site with ≤2 mismatches (in our notation, “x” represents infinity). At this default, 78% of candidate protospacers are discarded in human, compared to just 29% in *Drosophila*, reflecting the relative uniqueness and compactness of the latter (compare dark blue bars in Fig 2B).

**Table 2:** Species analysed by CRISPETa and for which off-target databases were compiled. Filtered protospacers are those passing default off-target and efficiency score cutoffs.

Species	Genome Version	Genome Size (Mb)	# PAMs (Total)	# filtered protospacers	Fraction filtered protospacers	Filtered protospacers / kb
Human	hg19	3140.751	298578412	20815659	0.07	6.6
Mouse	mm10	2785.489	145542344	23153774	0.16	8.3
Zebrafish	danRer10	1399.154	41970092	7321075	0.17	5.2
D. melanogaster	dm6	146.601	10684692	2909180	0.27	19.8
C. elegans	ce11	101.539	5027189	1079086	0.21	10.6

To estimate their efficiency in inducing double stranded breaks at their target sites, candidate protospacers are scored using the logistic regression measure of Doench et al[16]. This model was trained on experimental assays for 6085 and 1151 sgRNAs tiled across six mouse and three human genes, respectively. This score predicts sgRNA efficiency based on informative nucleotide preferences both within the core 20mer and in its immediate flanking nucleotides. Protospacers passing defined off-target and on-target thresholds are retained–henceforth referred to as “filtered protospacers”. In contrast to off-target filtering, efficiency score filters are more consistent across genomes, removing 60–70% of protospacers in the five genomes tested (light blue bars in Fig 2B, Table 2 and Supplementary S8 File). Together, off-target and efficiency score filters eliminate 96% of candidate protospacers in human (Fig 2B), but nevertheless yielding an average density of 6.6 usable protospacers per kilobase (Fig 2A). Comparison across species shows that there is markedly lower density of usable filtered protospacer sequences in vertebrates compared to invertebrates (Fig 2A). In general, and even after applying off-targets and score filters, the minimum deletion size constrained by sequence features alone is less than 150 bp for the majority of genomic regions (Supplementary S7 File).

In the final step, optimal sgRNA pairs are selected. First, all possible pairs of filtered protospacers are enumerated and ranked. Two ranking approaches are available: by combined efficiency score (default), or by length of deleted region. Ranking by score will tend to result in pairs that are more evenly distributed throughout the targeting region, but with a heterogeneous distribution of deletion sizes. Conversely, ranking by length favours shorter intervals within the constraints of the targeting design. Short segments may be more efficiently deleted [20], but will tend to be clustered into a smaller genomic region.

The top-ranked pairs, up to a user-defined maximum of *n*, are returned for each feature. In principle, a single high-scoring sgRNA may end up contributing to many or all of the highest-scoring pairs. To control this process, the “diversity” measure is used to control the maximum fraction of pairs containing a single sgRNA sequence (Table 1).

Finally, the user may specify constraints in sgRNA pair selection based on the plasmid construction method. Many plasmids employ the U6 promoter, which requires the sgRNA to commence with a “G”. For instance, the DECKO plasmid expresses two sgRNAs in tandem from U6 and H1 promoters, thus requiring the 5’ sgRNA to commence with G [4]. The “construction method” variable allows users to incorporate this constraint, specifically by ensuring that the first sgRNA commences with a natural or prepended G.

CRISPETa returns a ranked series of paired sgRNA constructs for each target. Sequences are output in a format suitable for immediate ordering from commercial oligonucleotide synthesis services. Summary statistics and figures are produced for each design job.

### Controlling CRISPETa performance by adjusting parameters

We tested the standalone pipeline using a set of 7000 human target genomic features compiled from a mixture of sources (see Materials and Methods). At default settings, CRISPETa returns successful, full depth (n = 10) designs for 68% of features, with a further 18% of partial depth (0<n<10) and 14% failures (“default” in Fig 2C, Table 3). We here define “full depth” to indicate the situation where all of *n* requested sgRNA pairs are successfully returned, and “partial depth” when the returned number is less than *n*. Performed on a workstation running CentOS6, 86.6 Gb of memory and 12 CPUs (Intel(R) Xeon(R) CPU E5649 @ 2.53GHz), the analysis took 44 minutes with a maximum RAM requirement of <100 MB.

**Table 3:** Benchmarking results. Analyses were performed on a set of 7000 regions composed of different human target types (see Methods for details). % full depth refers to the percent of targets receiving *n* = 10 sgRNA pair designs. % partial depth refers to targets receiving 0<*n*<10 designs. Designed targets refers to the total number of target features receiving full or partial depth designs.

Name	Non-default parameter	Wallclock Time (s)	% full depth	% partial depth	Mean pairs per target	Mean protospacer score	Mean pair score	Mean pair distance	Total sgRNA pairs	# Input targets	# Designed targets
Default		2639	68	18	8	0.52	1.04	1375	53760	7000	6024
Diversity0.1	v = 0.1	4748	0	86	1	0.62	1.25	1374	6024	7000	6024
Diversity 1	v = 1	4764	70	16	9	0.52	1.04	1375	54868	7000	6024
off1	t = 1,0,1,x,x	4869	83	9	9	0.57	1.15	1452	60959	7000	6417
off2	t = 1,0,2,x,x	4783	87	7	9	0.6	1.19	1451	62722	7000	6519
off3	t = 1,0,3,x,x	4802	88	6	9	0.6	1.20	1451	63514	7000	6564
off4	t = 1,0,4,x,x	4827	89	5	9	0.61	1.21	1450	63959	7000	6592
off5_1	t = 1,1,5,x,x	4883	90	5	9	0.6	1.21	1446	65030	7000	6695
pScore0.6	sp = 0.6	4878	63	23	8	0.53	1.06	1366	51488	7000	5974
pScore1	sp = 1	4691	29	41	7	0.6	1.20	1331	32674	7000	4881
pScore1.2	sp = 1.2	5318	9	42	2	0.67	1.33	1252	17226	7000	3587
PScore1.6	sp = 1.6	4658	0	7	0	0.83	1.66	1130	849	7000	513
design_2000	du = 2000 dd = 2000	9266	95	2	9	0.70	1.40	2874	67265	7000	6781

This benchmarking was repeated several times, in each case modifying a single parameter (Fig 2C and Table 3). As expected, strengthening the diversity requirement resulted in a drastic reduction of design success (“diversity0”), while a complete relaxation (“diversity1”) did not produce a substantial gain. Some improvement was observed when relaxing off-targeting, but this benefit is negligible after “off1” (allowing a single other match with two mismatches, (0:1, 1:0, 2:1, 3:x, 4:x). As expected, increasing the paired score threshold has a strong effect on design depth, particularly after 0.6 (“pScore0.6”) (the default is 0.4). The most dramatic improvement was observed when the length of the design region was increased to 2000 bp, boosting the fraction of successfully targeted regions from 70% to 95%. Thus, by adjusting these parameters, the depth of library designs can be optimised for each target set.

### Genome-scale deletion libraries for protein-coding and non-coding genomic elements in five species

**Fig 3:**
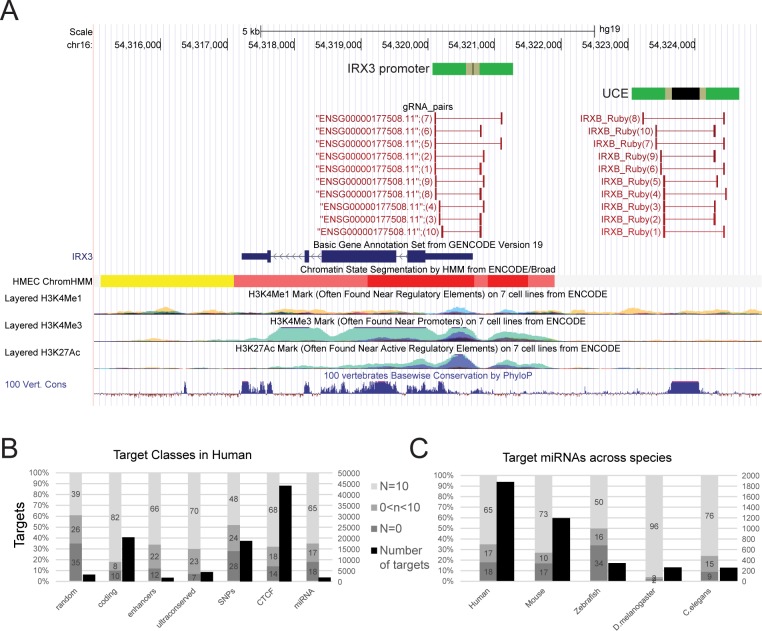
Genome-wide knockout libraries for entire classes of genomic elements in humans and other species. (A) An example of paired sgRNAs designed against the upstream ultraconserved element (UCE) and promoter of the human *IRX3* gene. *IRX3* lies on the antisense strand. The exact target regions are shown in black, flanked by the design regions in green. The ten sgRNA pairs for each are denoted by red bars. Integrated chromatin marks from the ENCODE project [26] are displayed below, in addition to PhyloP multispecies conservation scores [33]. Note the region of elevated conservation corresponding to the UCE. (B,C) Summary of paired sgRNA designs targeting entire classes of genomic elements. In each figure, the left scale and grey bars represent the design performance, as in Fig 2. The right scale and black bars indicate the total number of elements in each class. (B) shows a series of genomic element classes for human, while (C) shows designs for the entire set of annotated microRNA genes in five species. Designs were created with default settings; designs using “DECKO” construction method give identical results.

We next used CRISPETa to design knockout libraries for a variety of genomic element classes that cannot be targeted by traditional RNAi, either because their function is not thought to depend on RNA production (eg ultraconserved elements [UCEs])[21], or because their RNA product is too short (eg microRNAs) (see Table 4). We also created a collection of 3170 random intergenic target regions in human as a reference and for use as negative controls in screening projects. An example is shown in Fig 3A, created using the standard output of CRISPETa, where the *IRX3* gene promoter and an upstream ultraconserved element (UCE) were targeted.

**Table 4:** Pre-generated paired CRISPR libraries.

Feature Class	Species	Source	% full depth	% partial depth	Mean pairs per target	Mean sgRNA score	Mean pair score	Mean pair distance	Total sgRNA pairs	# Input targets	# Designed targets
Random intergenic	Human	Gencode hg37, UCSC, RefSeq	0.39	0.26	7	0.46	0.92	1864	15952	3170	2071
Protein coding gene promoters	Human	Gencode hg37	0.82	0.8	9	0.6	1.22	632	175116	20332	18304
Vista enhancers	Human	Vista enhancers browser hg37	0.66	0.22	8	0.50	1.00	2409	13236	1747	1522
Ultraconserved elements	Human	UCNE base hg37	0.70	0.23	8	0.49	0.99	985	35240	4351	4031
Flagged SNPs	Human	GWAS base hg37	0.48	0.24	8	0.48	0.96	1665	110135	18670	13526
CTCF	Human	Gencode hg37	0.68	0.18	8	0.53	1.06	795	335315	44056	37709
MicroRNA	Human	Mirbase hg38 > liftover to hg37	0.65	0.17	8	0.53	1.06	731	13788	1871	1540
MicroRNA	C.elegans	Mirbase ce11	0.76	0.15	9	0.55	1.09	713	2093	250	227
MicroRNA	Zebrafish	Mirbase z9	0.49	0.16	8	0.52	1.05	692	1944	337	221
MicroRNA	Mouse	Mirbase mm10	0.73	0.10	9	0.57	1.13	730	9268	1187	983
MicroRNA	D.melanogaster	Mirbase dm5	0.96	0.02	9	0.62	1.23	760	2508	256	251

The characteristics of these libraries are shown in Fig 3B and 3C and Table 4. Overall, 68% of features could be targeted at full depth, with an additional 18% at incomplete depth. We observe considerable heterogeneity in the design success across classes, with protein-coding gene promoters reaching a full depth for 82% of cases, compared to 39% in random intergenic regions. We expect these differences arise from the former’s high sequence uniqueness (decreasing off-target frequency) and high GC-content (increasing PAM density and predicted efficiency).

To compare performance across species, we created designs targeting the entire annotated catalogue of microRNA genes in human, mouse, zebrafish, *D*. *melanogaster* and *C*. *elegans* (Fig 3C). We observe considerably more efficient designs in non-mammalian species, likely reflecting their more compact, less repetitive nature. Nevertheless, at default settings we managed to create full or partial depth designs for 82% of human miRNA precursors, and this could likely be improved by altering design parameters.

The entire set of designs are available for download from crispeta.crg.eu. Overall these results demonstrate the practicality of creating large-scale paired sgRNA knockout designs across diverse genomic element classes.

### CRISPETa designs efficiently delete targets in human cells

**Fig 4:**
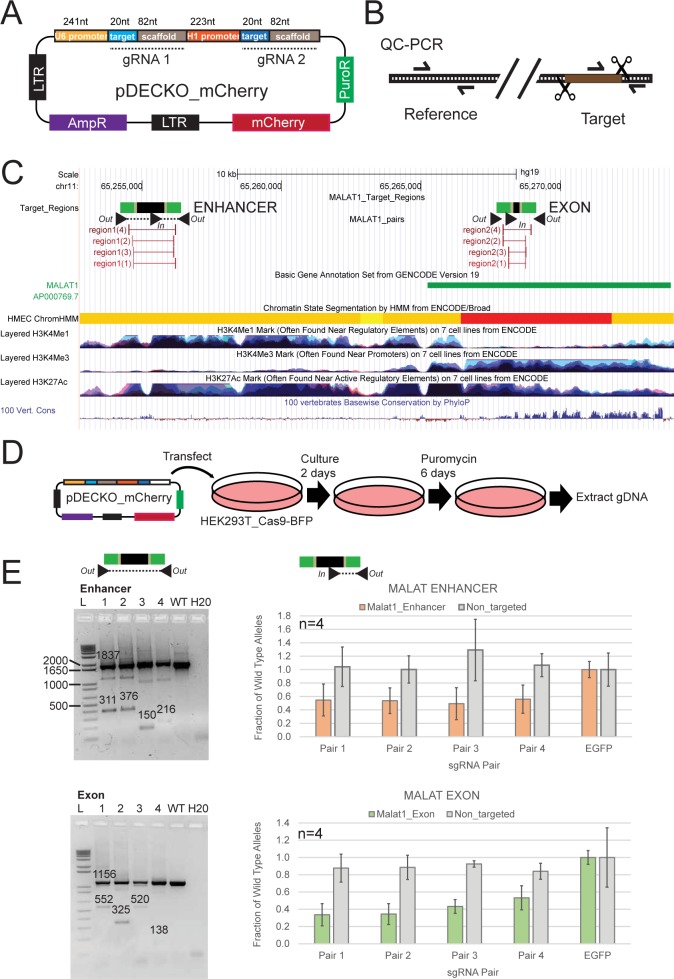
Measuring the deletion efficiency of paired sgRNA designs. (A) Structure of the pDECKO_mCherry vector. Note that the blue “target” regions contain protospacer sequences, and must be cloned as appropriate for each target region, while the grey “scaffold” regions are constant for all experiments. Note the presence of mCherry and puromycin selection markers. pDECKO_mCherry is compatible with lentivirus production or transfection. (B) Outline of the QC-PCR method for assessing deletion efficiency. Concentration of unmutated, wild-type target sites (using “In” and “Out” primers) is normalised to the reference amplicon, to control for template gDNA concentration. (C) The human *MALAT1* locus. The *MALAT1* lncRNA gene, shown in green, lies on the positive strand. The two selected target regions are shown: the conserved upstream enhancer-like region (note the overlap with H3K4Me1 and H3K28Ac modifications), and the exonic region. As before, target regions are shown in black, and sgRNA design regions in green. sgRNA pairs are represented by red bars, and genotyping primers as black arrowheads. (D) Overview of the experimental scheme. (E) Results for Enhancer region (upper panel) and Exon region (lower panel). Left: Agarose gel showing conventional genotyping results from bulk (unsorted) cells, with primers flanking the deleted region (primers out-out). 1–4 designate sgRNA pairs. “WT” indicates control cells transfected with pDECKO_mCherry targeting the EGFP sequence. Numbers on the gel refer to the expected size for the PCR amplicons. Right: QC-PCR results from four independent biological replicates (primers in-out). Y-axis shows the normalised fraction of unmutated, wild-type alleles, using primers amplifying the targeted region (orange/green), or a distal, non-targeted region (grey). Data were normalised to control cells transfected with pDECKO targeting EGFP. Error bars denote the standard deviation of four independent biological replicates. The differences between treated cells and control cells were statistically significant for all four sgRNA pairs, for both target regions (P<0.01, paired *t* test).

We next evaluated the performance of CRISPETa designs in an experimental setting. As an model, we focussed on the *MALAT1* locus, which expresses a potent oncogenic mono-exonic lncRNA [22]. In previous work, we managed to delete the *MALAT1* promoter using pairs of sgRNAs delivered by a lentiviral vector, pDECKO [4,22]. We have created an updated version, pDECKO_mCherry (hereafter referred to as “pDECKO” for brevity), carrying both antibiotic and fluorescence markers, into which designed sgRNA sequences can be rapidly cloned and expressed from independent promoters (Fig 4A). We also developed a streamlined protocol for cloning these vectors, DECKO2, described in detail in Supplementary S1 File.

We selected two target regions: a conserved upstream element with enhancer-like chromatin modifications (“enhancer”) and a region of conserved exonic sequence (“exon”) (Fig 4C). Each was submitted to CRISPETa, and from the resulting sgRNA designs we selected the three highest scoring pairs and one lower scoring pair (details can be found in Supplementary S5 File). HEK293T cells, stably expressing Cas9-BFP, were transfected with pDECKO, and selected by antibiotic resistance for 6 days, after which their genomic DNA (gDNA) was extracted (Fig 4D).

In order to observe genomic deletion, we used two distinct PCR-based methodologies. The first, non-quantitative approach allows one to verify the correct size of deleted regions using primers flanking the target region (Fig 4E, left panels). We used this “conventional” approach to genotype *MALAT1* deletions in a previous study [4]. The second approach, which we call “quantitative CRISPR PCR” (QC-PCR), allows one to estimate the deletion efficiency, in terms of percent of wild-type (uncut) alleles remaining in a cellular population (Fig 4B and 4E, right panels). In tests using mixtures of wild type and deleted alleles, QC-PCR could accurately estimate known concentrations (Supplementary S6 File). The primer configurations used by both approaches are shown as black arrowheads in Fig 4C and 4E.

We used both conventional genotyping and QC-PCR to investigate target region deletion in gDNA of transfected HEK293T cells (Fig 4D). Conventional PCR genotyping, using out-out primers, yielded amplification product sizes consistent with target site deletion for all pDECKO constructs, but not for control cells (Fig 4E, left panels). QC-PCR analysis, using in-out primers, of independent biological replicates showed loss of ~40% of enhancer target sites for each of the four sgRNA pair designs targeting the enhancer region (Fig 4E, top right). A non-targeted genomic region was not affected (“Non-targeted”). Higher efficiencies were observed for the exon-targeting constructs, yielding >60% efficiency for the top two sgRNA pairs (Fig 4E, bottom right). We did not observe a strong difference in the deletion efficiency between the four sgRNA pairs targeting the enhancer, although for the exon region, the lower-scoring two constructs displayed reduced efficiency. This underlines the value of using predicted efficiency scores in sgRNA selection, and supports the effectiveness of CRISPETa-predicted sgRNA pairs.

### Genomic deletion results in mutant RNA production

**Fig 5:**
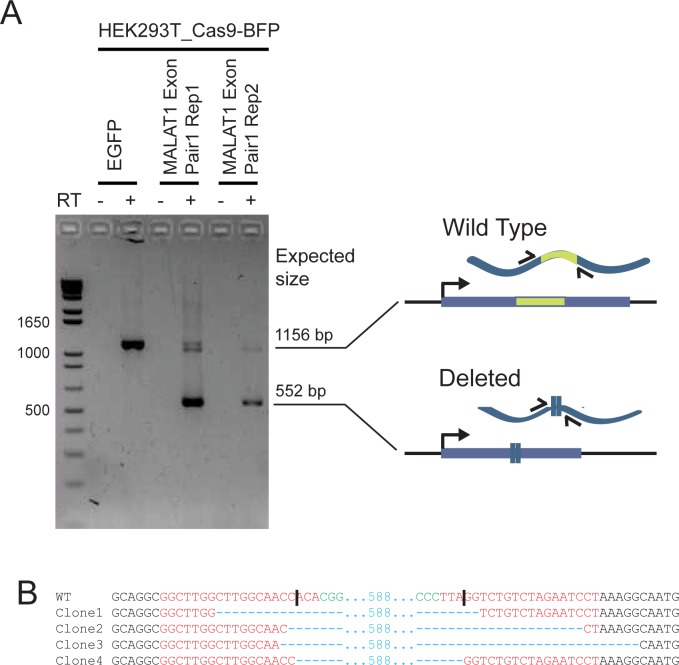
Production of truncated *MALAT1* RNA from mutated alleles. (A) RT-PCR was performed on RNA from bulk cells where MALAT1 exon region was deleted (sgRNA Pair 1, in two biological replicates), or control cells transfected with pDECKO targeting EGFP. Primers flanking the deleted region were used, and are expected to amplify fragments of the indicated sizes, depending on whether the RNA arises from a wild type or a deleted allele. Specificity was ensured by the exclusion of the reverse transcriptase enzyme in control reactions (“RT-”). (B) Sequencing analysis of mutant junctions of 4 of the colonies after TA cloning of the RT-PCR product. In red, region complementary to the sgRNA variable region; Green, PAM sequences; Blue, indel. Expected cut location is marked with vertical bar.

We next sought to verify that the engineered deletions in the *MALAT1* exon result in the expected changes to transcribed RNA. cDNA was generated from bulk cells treated with pDECKO vectors targeting MALAT1 exon. Given that not all cells have both alleles deleted, this sample should contain a mixture of RNA from both wild-type and mutated alleles. RT-PCR using primers flanking the targeted region amplified two distinct products, of sizes expected for wild-type and deleted sequence (Fig 5A). TA cloning and Sanger sequencing of individual cDNA clones revealed a variety of deletion sites around the expected position within the *MALAT1* exon (Fig 5B). Therefore, targeted deletions by CRISPETa are reproduced in the transcriptome, and may be used in future dissect RNA functional elements.

## Discussion

We have here presented a versatile and scalable design solution for CRISPR deletion projects. To our knowledge, CRISPETa is the first tool for selection of optimal sgRNA pairs. A key feature is its scalability, making it equally suitable for focussed projects involving single target regions, and screening projects involving thousands of targets. The user has a large degree of control over the design process, enabling projects to be optimised for target regions with diverse sequence uniqueness and GC content. On-target efficiency is predicted using the latest, experimentally-informed design algorithm, while running speed is boosted by an efficient off-target calculation.

A growing number of laboratories are adopting CRISPR deletion in their research for diverse applications, including modelling of human genetic disease [1], functional dissection of enhancer elements [3] or insulators [23], or loss-of-function studies on small or long noncoding RNAs [2]. In each case, it was necessary to manually design pairs of sgRNAs using available, single sgRNA design tools. There is clearly ample space to streamline this process. The second main application for CRISPR-deletion is for high-throughput loss-of-function screening studies, through the cloning of complex, pooled targeting libraries. These have enormous potential for the systematic identification of functional, non-coding genomic elements for the first time [3]. Manual design of paired sgRNAs for such projects is clearly out of the question. CRISPETa has been designed with both types of project in mind.

The QC-PCR technique presented here now allows one to quantify and compare the efficiency of CRISPETa designs. For the 8 sgRNA pairs in two regions that we tested, deletion efficiencies of ~40–60% were consistently observed. Given that DECKO gives rise to an approximately equal mixture of heterozygous and homozygous mutants [4], this would imply that over half of the cells in the mixture are being mutated. The induced deletions, when occurring within a transcribed region, are also observed in expressed RNA molecules. This is, to our knowledge, the first demonstration of the production of truncated RNA from an edited locus.

It should be noted that our understanding of on- and off-target sgRNA efficiencies is evolving rapidly. The Doench score used here is trained on a limited number of protein-coding genes, and it is likely that its scoring algorithm will be further refined in the near future. We plan to incorporate such improvements into CRISPETa as they become available. Users who wish to omit the on-target filter, may simply set the on-target score thresholds to zero. Similarly, to remove off-target filters, users may set all mismatch settings to infinity.

CRISPR enables us to study the function of non-coding genomic elements in their endogenous cellular context for the first time. The power of CRISPR-Cas9 genome-engineering lies both in its versatility, but also in its ready adaptation to large-scale screening approaches. The CRISPETa pipeline and experimental methods described here will, we hope, be useful for such studies.

## Materials and Methods

### Details of CRISPETa code

The pipeline is outlined in Fig 1A. As input, CRISPETa requires a standard BED6-format file describing one or more target regions of the supported genomes. Presently these are hg19 (human), mm10 (mouse), danRer10 (zebrafish), dm6 (*Drosophila*), ce11 (*C*. *elegans*). The webserver also directly accepts input as sequence, in FASTA format. Unstranded entries are assigned to the + strand, while those without identifiers are assigned a random ID. CRISPETa first defines design regions based on parameters *g*/*du*/*dd*/*eu*/*ed* (see Table 1 for full list of parameters) (Fig 1A and 1B), and extracts their sequences using the BEDtools *getfasta* function. Design regions are searched for canonical PAM elements (NGG) using a regular expression. For every such PAM, a total of 30 nucleotides (NNNN[20nt]NGGNNN) are stored. Protospacers containing the RNA Pol III stop sequence (TTTTT) are removed.

Next, candidate protospacers are searched against a precomputed, database-stored list of potential protospacers and their number of similar sequences with up to 4 mismatches, genome-wide (see “Off-target analysis” section, below). Matches (≤2 mismatches) to non-canonical (“NAG”) protospacers in any annotated protein-coding region are excluded from all analyses. By default, protospacers with one or more off-targets with ≤2 mismatch are discarded (this cutoff can be modified by the user through parameter *t*). Remaining protospacers are then compared with the positive and negative mask BED files using BEDtools *intersectBed*. Candidate sequences not fully overlapping the positive mask file, or overlapping the negative mask by one basepair, are tagged as “disfavoured”. Next, 30mer regions encompassing remaining protospacers, including disfavoured ones, are assigned an efficiency score (see below) between 0 and 1, and those above the score threshold (controlled by parameter *si*) are carried forward.

Next, candidate sequences are assembled into pairs and filtered. For each target region, all possible pairs of upstream and downstream candidates are generated. If pairs are designed for DECKO cloning (which utilizes the U6 promoter for the 5’ sgRNA gene, controlled by *c*), an additional step is applied: sgRNA pairs, where one of the pairs commences with G, are reordered as necessary such that the first sgRNA starts with G; for pairs where neither commences with G, an additional G is prepended to the first sgRNA[24]. It should be noted that this “DECKO construction” mode thus results in oligonucleotide libraries that vary in length by one nucleotide. A combined score for the resulting pairs is computed. By default, this is the sum of the two individual sgRNA scores, but users may choose to define the pair score as the product of individual scores (parameter *sc*). Pairs are now filtered with a pair score threshold, and ranked first by mask score and then by pair score (or, optionally, reverse ranked by distance, using parameter *r*). An optional “diversity” cutoff can be used to remove pairs such that no individual candidate sequence appears in more than a given fraction of returned pairs (parameter *v*). Finally the program returns the top ranked pairs up to the maximum number specified by the user, *n*.

CRISPETa is implemented in Python and available for download from git-hub and the CRISPETa web-server (see availability below).

### Target features and mask files

All target sets and mask files were prepared in BED format, and obtained in April 2016. Coding genes were obtained from the Gencode v19 annotation, filtered for the “protein_coding” biotype [25]. CTCF binding sites for GM12878 cells were downloaded from ENCODE data hosted in the UCSC Browser [26]. Enhancers were obtained from Vista [27]. Pre-miRNAs were obtained from miRBASE [28]. Disease-associated SNPs were obtained from the GWAS database (http://www.ebi.ac.uk/gwas/api/search/downloads/full). Ultraconserved regions were obtained from UCNEbase [21]. For human positive and negative masks we used DNaseI hypersensitive sites identified through genome-wide profiling in 125 diverse cell and tissue types by the ENCODE consortium [29] and RepeatMasker repetitive regions [30], respectively. To generate random intergenic locations, the entire span of all Gencode v19 genes (both coding and noncoding, introns and exons), in addition to 100 kb up- and downstream, were subtracted. Random locations were selected within the remaining regions.

### Off-target analysis

Off-target analysis was performed using Crispr-Analyser [19]. We searched for all canonical PAM regions (NGG) in the genome and stored the 20nt that precedes each. Then using “search” and “align” options we obtained the number of off-targets with 0,1,2,3 and 4 mismatches for each unique 20mer. A second step was performed to remove protospacers with potential off-targeting in coding regions: for each genome, all 20mers followed by NGG were mapped using BWA mapper against sequences of 20nt followed either by NGG or NAG in all annotated protein-coding regions [31]. Those 20mers having alignments with ≤2 mismatches were removed in order to avoid potential off-targets in coding regions. Filtered 20mers were stored in a MySQL database. Precomputed files containing this information for various genomes can be directly downloaded (see “CRISPETa availability” section). Downloadable files contain 6 comma-separated fields in this order: sequence of the sgRNA without the PAM sequence and the number of off-targets with 0,1,2,3, and 4 mismatches for this sgRNA. These files can be used as input for CRISPETa-MySQL module to generate the MySQL database.

### sgRNA scoring algorithm

CRISPETa uses the scoring method developed by Doench et al [16], based on an experimentally trained logistic regression model employing 72 sequence features. The code was downloaded from http://www.broadinstitute.org/rnai/public/analysis-tools/sgrna-design-v1.

### Benchmarking

A test target set was assembled from 1000 randomly-selected elements from each of the individual target annotations, for a total of 7000. Benchmarking analyses were run on a workstation running CentOS6, 86.6 Gb of memory and 12 CPUs (Intel(R) Xeon(R) CPU E5649 @ 2.53GHz).

### CRISPETa availability and webserver

CRISPETa can be run through the web-server (http://crispeta.crg.eu) or locally. The software runs on python2.7. In order to run CRISPETa locally two additional programs are required: BEDtools and MySQL. Source code to run locally can be found at git-hub (https://github.com/guigolab/CRISPETA) and also at the “Get CRISPETa” section of the web-server. Source code consist of two scripts: CRISPETA.py that execute the main pipeline described above, and crispeta_mysql.py that helps users to create the off-target MySQL database. Two other files can be found within the source code: func.py that contains all functions necessary to execute the two main scrips, and config.py that stores the information needed to login to MySQL.

### Molecular cloning of pDECKO paired sgRNA vector

We used a modified version of our previously-described protocol for the creation of pDECKO_mCherry vectors expressing pairs of sgRNAs, DECKO2 [4]. A detailed protocol is available in Supplementary S1 File, as well as on CRISPETa webpage. Selected sgRNA pairs were converted to overlapping series of 6 oligonucleotides using a custom design spreadsheet (available as Supplementary S2 File). All described plasmids are available from Addgene.org under plasmid numbers 78534–78545.

### QC-PCR assay

gDNA was extracted with GeneJET Genomic DNA Purification Kit (Thermo Scientific) and quantitative real time PCR (qPCR) from 1.6 ng of purified gDNA was performed using Lightcycler 480 SYBR Green master kit (Roche) on a LightCycler 480 Real-Time PCR System (Roche). Primer sequences can be found in Supplementary S3 File. Target sequence primers (TFRC_B out F / TFRC_B in R, Enhancer in F / Enhancer out R for enhancer, Exon in F / Exon out R for exon) were normalised to primers GAPDH F/R amplifying a distal, non-targeted region. Another non-targeting primer set, LdhA F/R were treated in the same way. Data were normalised using the ΔΔCt method [32], incorporating primer efficiencies. The latter were estimated using a dilution series of gDNA, and efficiency calculated by the slope of the linear region only (Supplementary S4 File). We noted a decrease in efficiency at high template concentrations.

For testing the accuracy of the QC-PCR method, we used genomic templates containing known proportions of a target allele from our previous study [4]. Genomic DNA from a heterozygous clone for *TFRC* gene of the human, diploid cell line HCT-116, was used, combined with WT gDNA in controlled proportions.

## Supporting Information

S1 File Extended DECKO2 cloning protocol. (DOCX)

S2 File Design spreadsheet for creating DECKO2 oligonucleotides. (XLSX)

S3 File Oligonucleotide sequences. (DOCX)

S4 File Estimation of QC-PCR primer efficiencies. (PDF)

S5 File Details of *MALAT1* sgRNA pairs. (XLSX)

S6 File Assessing the accuracy of the QC-PCR method. We tested the accuracy of QC-PCR using gDNA templates containing known proportions of a target allele. In a previous study, we generated a mutant clone of the human, diploid cell line HCT-116 [4], where one copy of the *TFRC* gene promoter was deleted by DECKO. This was verified by careful genotyping. Thus *TFRC* promoter must be at 50% concentration in gDNA from this clone. By mixing this gDNA with wild type HCT-116 cells’ gDNA in varying proportions, we created a dilution series of known *TFRC* promoter concentrations (*x* axis). We used “In-Out” primers of known efficiency to amplify either the *TFRC* promoter region (yellow bars, primers “TFRC_B out F” and “TFRC_B in R” in Supplementary S3 File) or a non-targeted distal region (grey bars, primers “LdhA F/R”). Experiments were performed on three replicate dilution series from the same starting samples of gDNA. QC-PCR experiments were carried out as described in the Materials and Methods. Comparison of the measured wild type allele concentration, and the true concentration, lead us to conclude that QC-PCR is suitable for assaying CRISPR deletion efficiency.
(PDF)

S7 File Distances to closest protospacers boxplot. For every filtered protospacer, the distance to the next nearest filtered protospacer is calculated. Boxplots shows the distribution of these distances. Thick bar indicates the median, and boxes indicate the interquartile range.
(PDF)

S8 File Filtered protospacer scores density plot. Density distribution of filtered protospacers scores computed with RuleSet1 algorithm (“Doench Score”, [16]). Vertical lines indicate the median for each distribution.
(PDF)
