# Supplementary material for: Scalable Design of Paired CRISPR Guide RNAs for Genomic Deletion
Source: PLoS Comput Biol. 2017 Mar 2;13(3):e1005341. doi: 10.1371/journal.pcbi.1005341 (PMC5333799; doi:10.1371/journal.pcbi.1005341)
Supplement: S1 File — (DOCX) [file pcbi.1005341.s001.docx]

PROTOCOLS DECKO2 CLONING

Carme Arnan ([carme.arnan@crg.eu](mailto:carme.arnan@crg.eu))

Rory Johnson (Lab Roderic Guigó)

July 2016

Version 6

**Overview of DECKO Cloning**

This document describes the cloning of individual targeting plasmids (as opposed to library cloning). DECKO employs a 2-step cloning methodology. Cloning is based on the Gibson assembly method, which avoids conventional ligation and enables multi-fragment assembly. In Step 1, multiple oligonucleotides are assembled together with the Backbone plasmid to create an intermediate plasmid. In Step 2, a constant “Insert-2” fragment is inserted within the new sequence created in the previous step. Insert-2 sequence can be amplified by PCR using oligos and Template plasmid described below. The time required for the complete cloning is about 5 days.

**Why use DECKO2?**

DECKO2 is a cloning protocol that has been optimised for cloning individual CRISPR targeting constructs. This is due to two improvements: first, Insert-1 is assembled from a series of six shorter, overlapping oligonucleotides (two of which are invariant for all targets), thereby reducing oligonucleotide synthesis costs by ~70%. Second, conventional ligation is eliminated, with both cloning steps relying on Gibson assembly. Note that, for pooled cloning of complex targeting libraries, the original DECKO protocol should be used (Aparicio-Prat et al, PMID26493208). The pDECKO_mCherry vector backbone is equally appropriate for original DECKO and DECKO2 cloning.


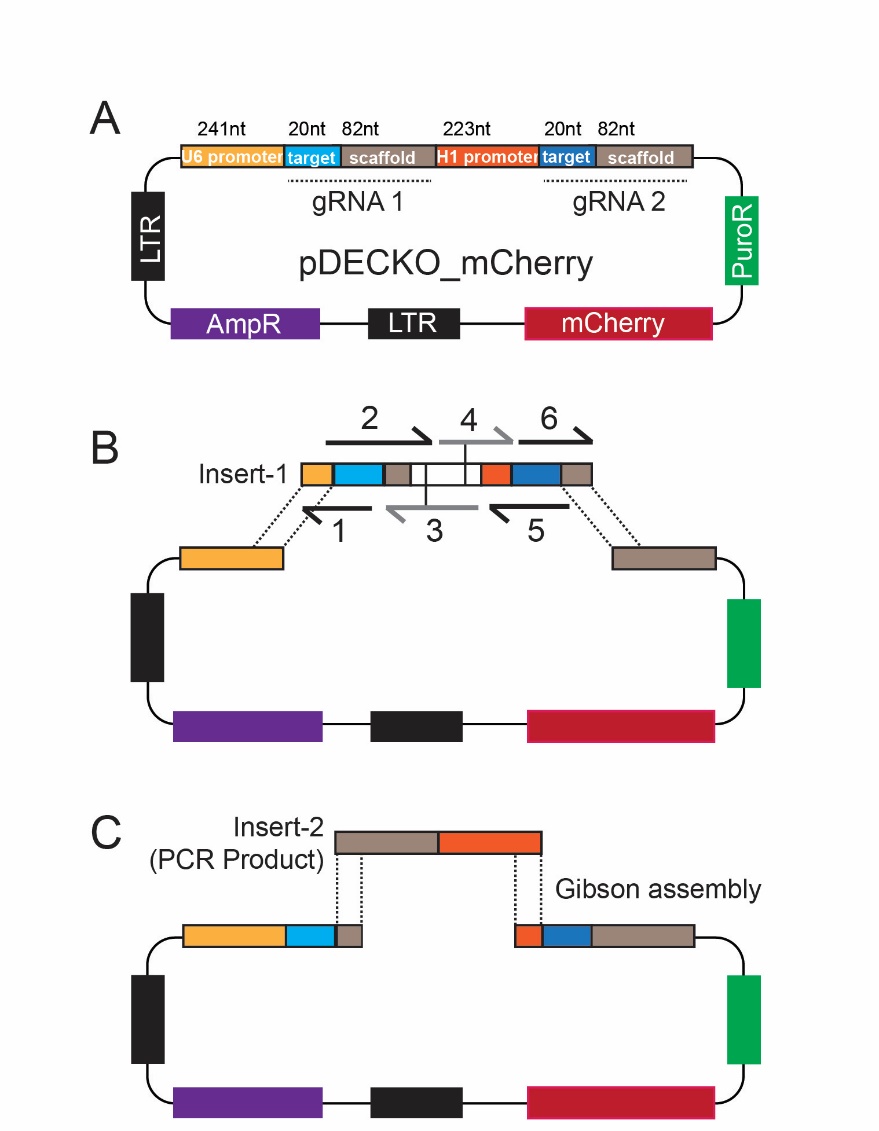


Fig.1. Diagram showing: (A) final pDECKO_mCherry construct (B) Step1 cloning (multiple oligo assembly) (C) Step 2 cloning (constant fragment assembly)


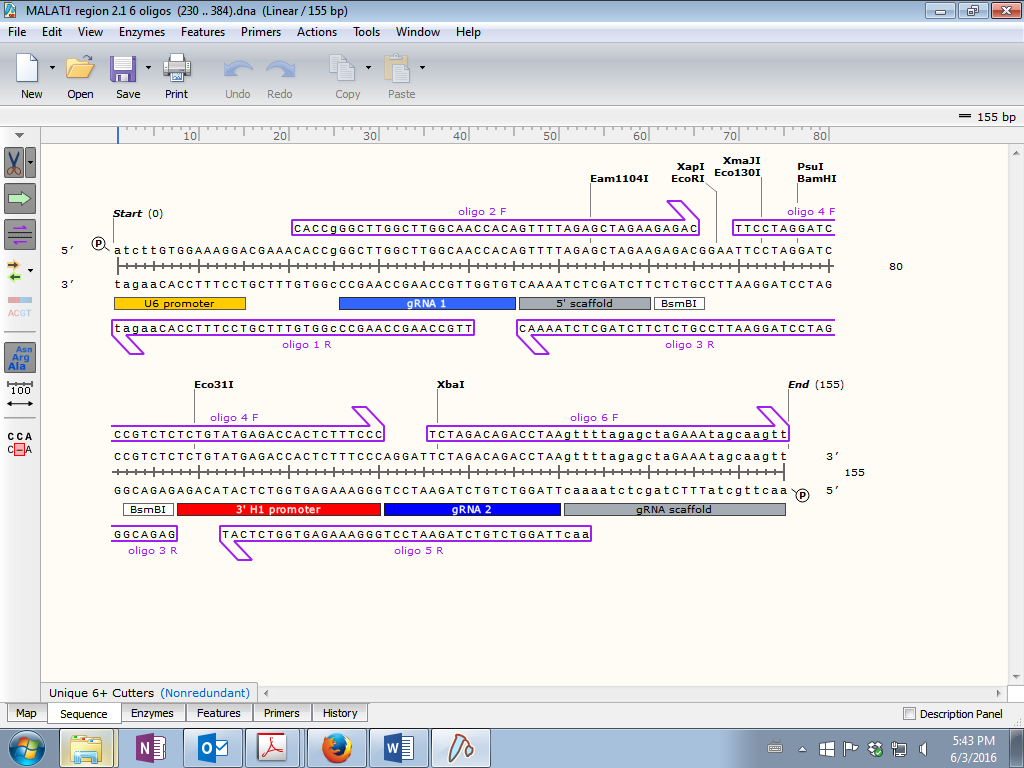
Fig.2 Detail of 6 oligo assembly example.

**Materials Needed**

- **6 oligo design spreadsheet**: This tool designs the 6 required oligos for assembly, based on the user-defined sgRNA targeting sequences.
- **Plasmids**:

1. Backbone plasmid: pDECKO_mCherry (available from Addgene: 78534)
2. Template plasmid (for insert-2 amplification)

Can use any complete pDECKO plasmid (for instance the ones cited in the paper Aparicio-Prat et al. BMC genomics 2015, available from Addgene)

- **Oligos**:

Oligos for Step1 cloning (oligo1_R, oligo2_F, oligo3_R, oligo4_F, oligo5_R, oligo6_F) *Note that oligos 3 and 4 are constant for all the constructs*.

Oligo3_R:GAGACGGGATCCTAGGAATTCCGTCTCTTCTAGCTCTAAAAC

Oligo4_F:TTCCTAGGATCCCGTCTCTCTGTATGAGACCACTCTTTCCC

Oligos for Step2 cloning: these oligos are used for the amplification of Insert-2 fragment using the Template plasmid.

C557F: GTTTTAGAGCTAGAAATAGCAAG

C557R: GTGGTCTCATACAGAACTTATAAG

Oligos for colony PCR and sequencing (are complementary to Backbone plasmid outside cloning region, and can be used for sequence verification)

C542F: GTACAAAATACGTGACGTAG

C542R: ATGTCTACTATTCTTTCCCC

- **Restriction endonucleases**:

BsmbI (Thermo Fisher ref.ER0451)

- **Gibson mix**:

Can be obtained from commercial source (Biolabs Gibson Assembly Master mix ref.E2611S) or prepared home-made following the protocol described in *Gibson DG et al. Nat. Methods 2009*.

- **Competent cells**:

Should be RecA- (for instance Stbl3) in order to prevent recombination of LTRs.

**DESIGN AND CLONING OF pDECKO PLASMIDS**

Design of the gRNA variable sequences using the software: CRISPETA (Pulido et al, Manuscript in preparation). Also see the webpage: <http://crispeta.crg.eu>.

Use the output of the program, together with the Design Spreadsheet for the 6 oligonucleotide design for DECKO2 cloning (available at http://crispeta.crg.eu/protocols). *Note that the first oligo needs to be reverse and the oligos are alternated (reverse-forward-reverse-forward-reverse-forward) for correct annealing in the Gibson reaction*.

If you do not have access to CRISPETA, we recommend use of CRISPR Design too from MIT (<http://crispr.mit.edu>) for sgRNA design.

**Day1**

STEP1 CLONING “6 OLIGOS ASSEMBLY” STRATEGY

1,1 Backbone plasmid digestion

Digestion with BsmbI (Thermo Fisher) to open pDECKO_mCherry (Addgene ref. 78534).

*Note: recommend to do at least 3 digestions in parallel and combine them to have enough plasmid concentration. In order to obtain a clean digestion it is recommended to use pure plasmid preferably from midi or maxiprep.*

5 ug plasmid

5ul Tango 10x buffer

2.5 ul DTT 20 mM

H20 up to 49 ul

1 ul BsmbI

Vf=50 ul

Digestion 2h at 37ºC (Heat block).

Load a 0.8% agarose gel (40’ at 100V). In the gel you should see 2 bands (8Kb and 2 Kb), the larger band should be cut and gel purified (elution in 30 ul H_2_0).


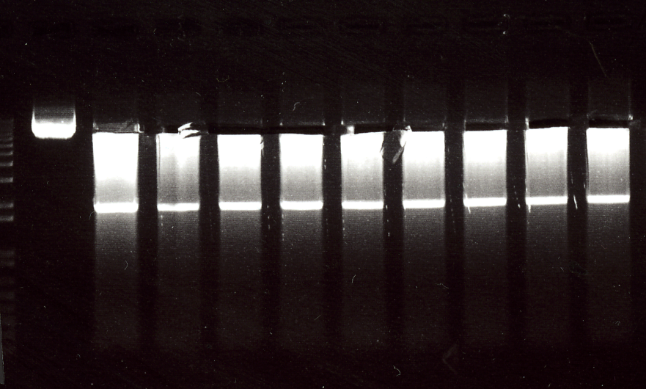


Undigested plasmid

8kb

2kb

BsmbI digestion

1.2 Gibson cloning using 6 oligos

1 ul 1uM oligo1_R

1ul 1 uM oligo2_F

1ul 1uM oligo3_R

1ul 1uM oligo4_F

1 ul 1uM oligo5_R

1 ul 1uM oligo6_F

(80-200 ng) BsmBI digested plasmid (up to 4 ul plasmid)

10 ul Gibson 2x (home-made CRG protein facility or commercial)

Vf=20 ul

Gibson reaction: Incubate 1h 50ºC (PCR machine)

1.3 Transformation

Transform 2ul into 50 ul z-Stbl3 competent cells

Incubate 5’ on ice and plate everything in LB+Amp and incubate 37ºC O/N

*Note: z-Stbl3 is a Rec A- strain to prevent recombination of Lentivirus plasmids and competents are prepared with Mix and Go E.coli Transformation Kit (ref. T3001 from Zymo Research)*.

**Day2**

1.4 Colony PCR

To check for positive clones. Check 6 clones per construct, in addition to empty control (backbone plasmid #739) and H20. We expect a band of size around 450bp.

Thermo pol buffer 10x 2ul (1)Initial denaturation 95ºC 30’’

Oligo C542F 10 uM 0.4ul   (2)Denaturation           95ºC 30’’

Oligo C542R 10 uM               0.4ul                 (3)Annealing               50ºC  1’

dNTP 10 mM                          0.4ul                 (4)Polymerization         68ºC  1’

H20                                         14.7ul                 (5)Go to 2 for 29 times

Taq polymerase (Biolabs)        0.1ul                 (6) Final extension      68ºC  5’

Template colony (into 20 ul H20) 2ul        (7) 4ºC forever Vf= 20 ul

Prepare a 1.5% agarose gel to check the clones. Run the gel at 100V 40’.


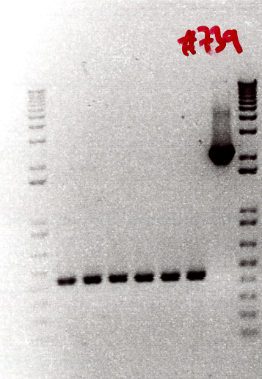


450 bp

C

2Kb

*Use primers C542F: GTACAAAATACGTGACGTAG and C542R: ATGTCTACTATTCTTTCCCC and use as a control the backbone plasmid #739 (Lenti-guide puro: Addgene ref. 52963)*

1.5 Precultures

Prepare some pre-cultures (2-4) from positive clones. 1 colony into 4ml of LB+Amp and growth O/N at 37ºC.

**Day3**

1.6 Minipreps

- Centrifuge the pre-cultures 5’ at 5000 rpm. Discard the supernatant.
- Prepare minipreps (2 minipreps per construct and freeze down pellets for the remaining 2). Final elution in 50 ul H20. Measure concentration and use the more concentrated ones for digestion.

*At this step the plasmids can be send to sequencing for checking the correct assembly but we recommend to continue with the following cloning step and check the final sequence at the end.*

STEP2 CLONING OF CONSTANT PART

2.1 Intermediate plasmid digestion

Digest intermediate plasmid with BsmbI to linearise it and clone the missing part (second scaffold and H1 promoter) by Gibson assembly.

5 ug plasmid

5ul Tango 10x buffer

2.5 ul DTT 20 mM

H20 up to 49 ul

1 ul BsmbI

Vf=50 ul

Digest 2h at 37ºC (Thermo Block).

*Note: After digestion you can gel-purify the band (but with low efficiency of recovery). It is recommended to do a dephosphorylation step (to prevent religations) and purify the DNA trough a column (elution in 30 ul of H20).*

2.2 Alkaline phosphatase digestion

- Add 5 ul of Alkaline phosphatase (Fast enzyme) 1U/1ug
- Digest 15’ at 37ºC (Thermo block)
- Stop digestion at 75ºC for 5’
- Do a buffer exchange in column and elution in 30 ul of H20.
- Measure concentration of plasmid by Nanodrop.

2.3 PCR of constant part (2^nd^ Scaffold+H1 promoter)

*Use as a template any complete pDECKO plasmid. Recommend to do 2 amplifications in parallel to have more material.*

*Note: primer sequences are C557F: GTTTTAGAGCTAGAAATAGCAAG C557R: GTGGTCTCATACAGAACTTATAAG*

10xbuffer2 2.5ul (1) Activation 94ºC 1´30”

dNTPs 10mM 0.5ul (2) Denaturation 94ºC 30”

oligo C557_F 10 uM 1ul (3) Annealing 50ºC 30”

oligo C557_R 10 uM 1ul (4) Extension 72ºC 1’

H20 18.5ul (5) Go to step 2 for 29 cycles

Expand polymerase 0.5ul (6) Additional extension 72ºC 10’

Template 1ul (plasmid diluted 1/100) (7) Cool down 4ºC

Vf= 25ul

- Do a gel of 1.5% agarose and run 40’ at 100V. Expected band size around 250 bp.
- Gel purify the amplified band (elution in 30 ul d’H20).
- Measure the concentration by Nanodrop.

2.4 Gibson cloning

(100-200 ng) BsmbI digested plasmid (up to 9 ul plasmid)

1 ul PCR insert (25-50 ng)

10 ul Gibson 2x

VF=20 ul

Gibson reaction: Incubate 1h 50ºC (Thermoblock)

2.5 Transformation

Transformation 2 ul Gibson into 50 ul Stbl3. Incubate 5’ on ice and plate everything in LB+Amp (O/N 37ºC).

**Day 4**

2.6 Colony PCR (same conditions as in step 1.4)

Colony PCR using primers C542F/R and intermediate plasmid as a negative control. Check 6 colonies each. We expect a band of size around 700 bp.


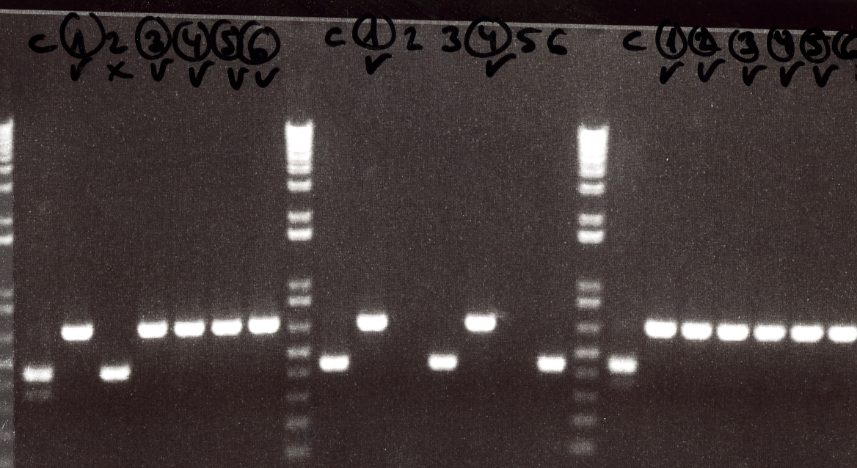


700 bp

C

450 bp

Prepare 4 pre-cultures per each positive construct (4 ml LB+Amp) O/N 37ºC

**Day 5**

2.7 Minipreps and sequencing

- Centrifuge pre-cultures 5’ at 4000 rpm (discard supernatant).
- Minipreps of 2 clones per construct (freeze down the remaining 2 pellets). Elution in 50 ul H20.Measure concentration by Nanodrop.
- Send to Sanger sequencing with primers C542F/R.
