# Supplementary material for: Scalable Design of Paired CRISPR Guide RNAs for Genomic Deletion
Source: PLoS Comput Biol. 2017 Mar 2;13(3):e1005341. doi: 10.1371/journal.pcbi.1005341 (PMC5333799; doi:10.1371/journal.pcbi.1005341)
Supplement: S3 File — (DOCX) [file pcbi.1005341.s003.docx]

**Oligonucleotide Sequences**

| Primer | Sequence |
| --- | --- |
| TFRC_B out F | CGCAAAGCACTCCGCTAGT |
| TFRC_B out R | ACACGAGGGTCGGTGTAGTTC |
| TFRC_B in R | GAAATGTACGTGCAGGATGGA |
| Enhancer out F | GGAAGGGGCCTCTTGAGTT |
| Enhancer out R | CATCTCAGCCCTTGTTATCCTG |
| Enhancer in F | GCTGGGGAATCCACAGAGAC |
| Exon out F | CCCAGGTGCTACACAGAAGTG |
| Exon out R | CTCTTCCCTGTTAAGACCATCCC |
| Exon in F | GGGGGCAAAATATGTTTTCAGT |
| LdhA F | TGGGCAGTAGAAAGTGCAG |
| LdhA R | TACCAGCTCCCACTCACAG |
| GAPDH F | CCGGGAAGGAAATGAATGG |
| GAPDH R | GAGCGCAGGGTTAGTCAC |
