# Supplementary material for: Scalable Design of Paired CRISPR Guide RNAs for Genomic Deletion
Source: PLoS Comput Biol. 2017 Mar 2;13(3):e1005341. doi: 10.1371/journal.pcbi.1005341 (PMC5333799; doi:10.1371/journal.pcbi.1005341)

Primer efficiency calculations for experiments on TFRC promoter.

	Amplyfication efficiency : E = 10^(-1/slope)		
	% efficiency = (E-1)*100%		
Primer	slopes (m)	E	Efficiency (%)
TFRC_B out F/TFRC_B in R	-3.22	2.04536742	105
GAPDH F/R	-3.27	2.02005019	102
LdhA F/R	-3.22	2.04395923	104

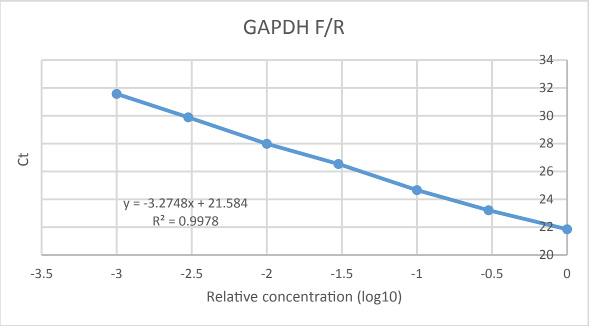

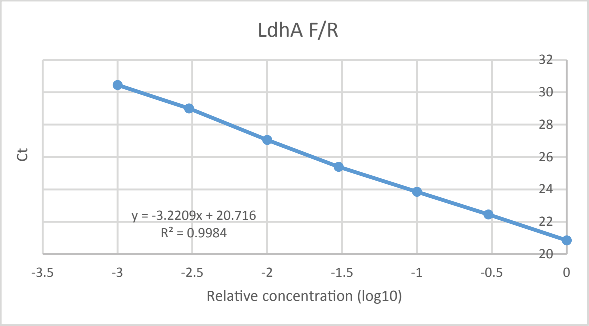

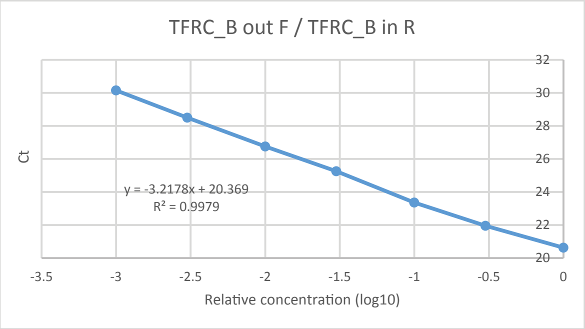

Primer efficiency calculations for experiments on MALAT1 locus.

Efficiency estimated from log linear fit to the red, linear region.

	Amplification efficiency : E = 10^(-1/slope)		
	% efficiency = (E-1)*100%		
Primer	slope (m)	E	Efficiency (%)
Enhancer in F / out R	-2,99	2,15997219	116
Exon in F / out R	-3,27	2,02213617	102
GAPDH F/R	-3,09	2,10680624	111
LdhA F/R	-2,9	2,21221629	121

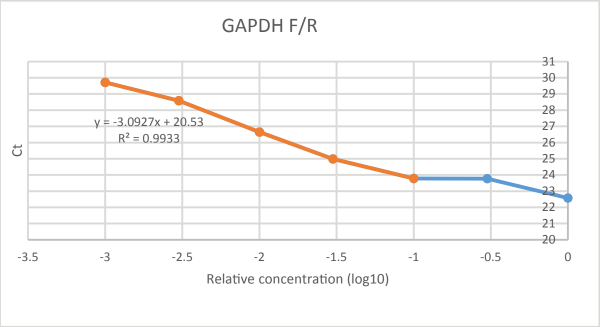

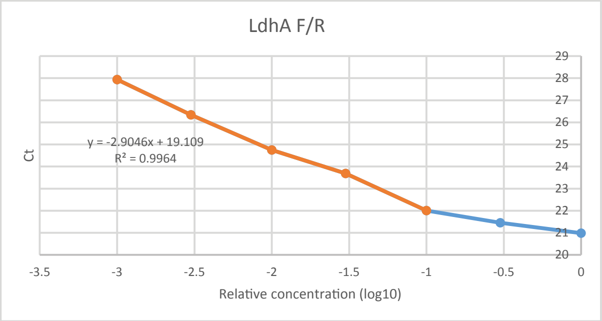

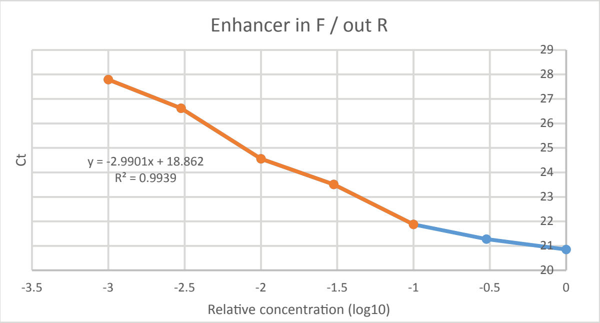

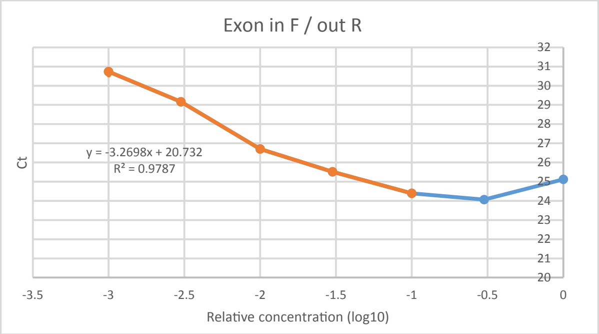

Supplement: S4 File — (PDF) [file pcbi.1005341.s004.pdf]
