# Supplementary figures and images for: Scalable Design of Paired CRISPR Guide RNAs for Genomic Deletion
Source: PLoS Comput Biol. 2017 Mar 2;13(3):e1005341. doi: 10.1371/journal.pcbi.1005341 (PMC5333799; doi:10.1371/journal.pcbi.1005341)

## TFRC Promoter

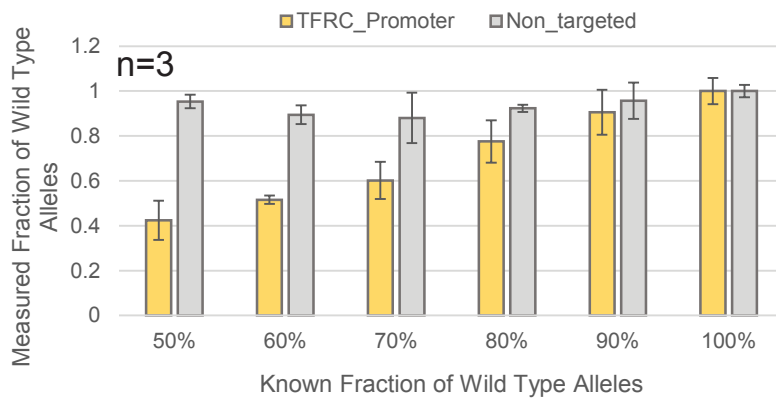

Supplement: S6 File — We tested the accuracy of QC-PCR using gDNA templates containing known proportions of a target allele. In a previous study, we generated a mutant clone of the human, diploid cell line HCT-116 [4], where one copy of the TFRC gene promoter was deleted by DECKO. This was verified by careful genotyping. Thus TFRC promoter must be at 50% concentration in gDNA from this clone. By mixing this gDNA with wild type HCT-116 cells’ gDNA in varying proportions, we created a dilution series of known TFRC promoter concentrations (x axis). We used “In-Out” primers of known efficiency to amplify either the TFRC promoter region (yellow bars, primers “TFRC_B out F” and “TFRC_B in R” in Supplementary S3 File) or a non-targeted distal region (grey bars, primers “LdhA F/R”). Experiments were performed on three replicate dilution series from the same starting samples of gDNA. QC-PCR experiments were carried out as described in the Materials and Methods. Comparison of the measured wild type allele concentration, and the true concentration, lead us to conclude that QC-PCR is suitable for assaying CRISPR deletion efficiency. (PDF) [file pcbi.1005341.s006.pdf]

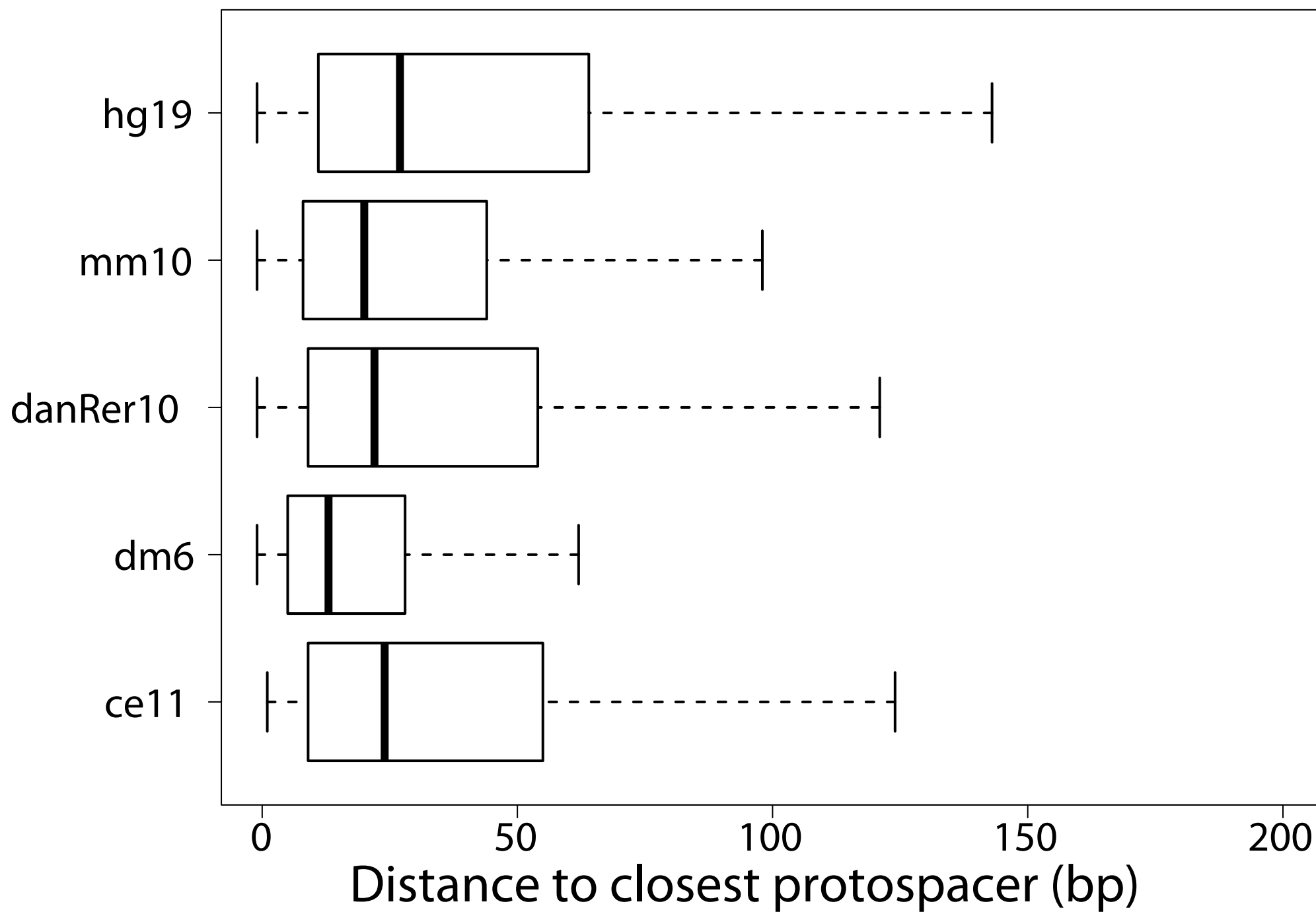

Supplement: S7 File — For every filtered protospacer, the distance to the next nearest filtered protospacer is calculated. Boxplots shows the distribution of these distances. Thick bar indicates the median, and boxes indicate the interquartile range. (PDF) [file pcbi.1005341.s007.pdf]

# Filtered protospacer scores density

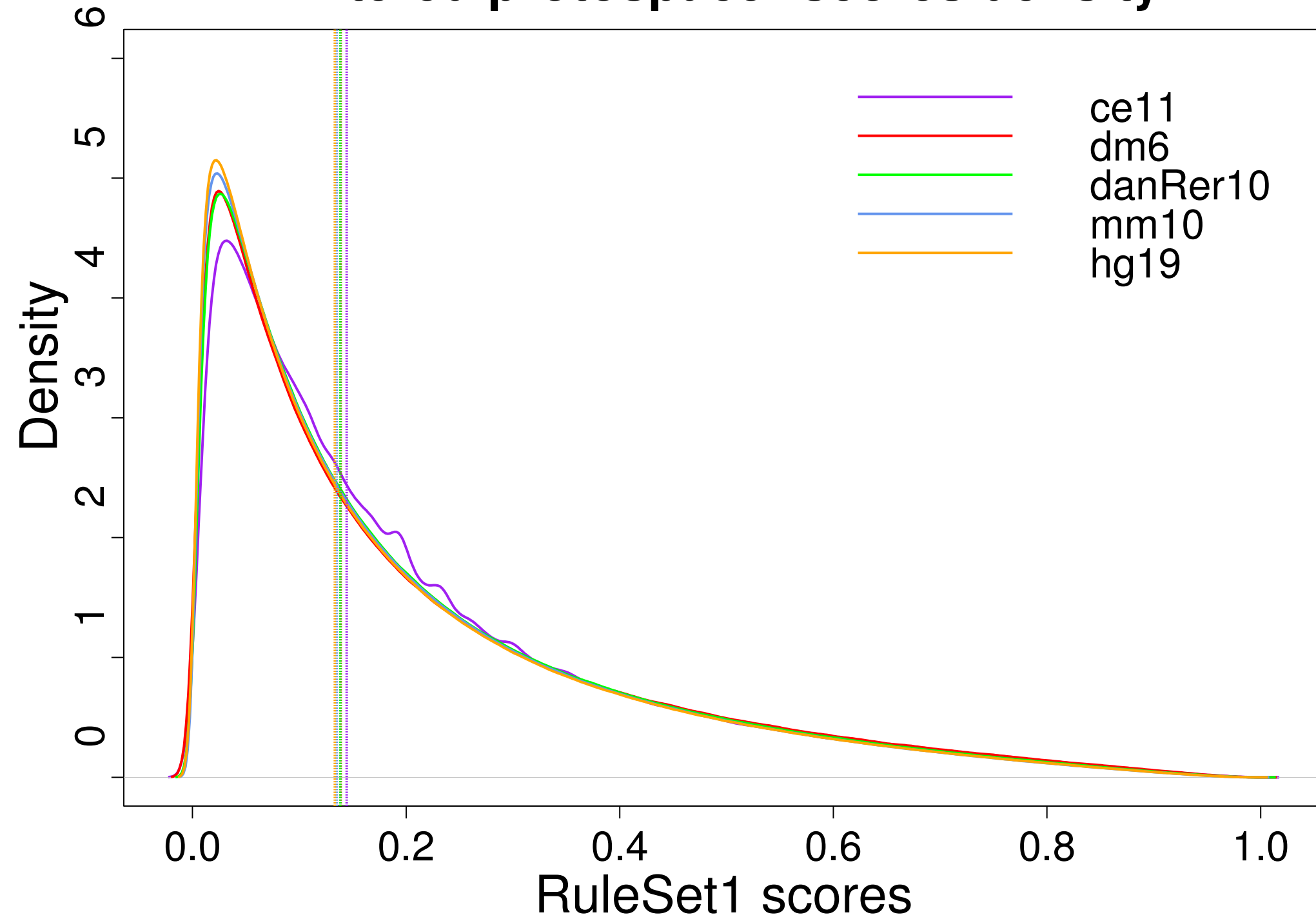

Supplement: S8 File — Density distribution of filtered protospacers scores computed with RuleSet1 algorithm (“Doench Score”, [16]). Vertical lines indicate the median for each distribution. (PDF) [file pcbi.1005341.s008.pdf]
